# Metabolic Cost of Activation and Mechanical Efficiency of Mouse Soleus Muscle Fiber Bundles During Repetitive Concentric and Eccentric Contractions

**DOI:** 10.3389/fphys.2019.00760

**Published:** 2019-06-21

**Authors:** Koen K. Lemaire, Richard T. Jaspers, Dinant A. Kistemaker, A. J. “Knoek” van Soest, Willem J. van der Laarse

**Affiliations:** ^1^Amsterdam Movement Science, Vrije Universiteit Amsterdam, Amsterdam, Netherlands; ^2^Laboratory for Myology, Amsterdam Movement Science, Vrije Universiteit Amsterdam, Amsterdam, Netherlands; ^3^Department of Physiology, Amsterdam Cardiovascular Sciences, Amsterdam University Medical Center, Amsterdam, Netherlands

**Keywords:** mechanical efficiency, blebbistatin, mouse soleus muscle, oxygen consumption, cross-bridge cycling, muscle activation

## Abstract

Currently available data on the energetics of isolated muscle preparations are based on bouts of less than 10 muscle contractions, whereas metabolic energy consumption is mostly relevant during steady state tasks such as locomotion. In this study we quantified the energetics of small fiber bundles of mouse soleus muscle during prolonged (2 min) series of contractions. Bundles (*N* = 9) were subjected to sinusoidal length changes, while measuring force and oxygen consumption. Stimulation (five pulses at 100 Hz) occurred either during shortening or during lengthening. Movement frequency (2–3 Hz) and amplitude (0.25–0.50 mm; corresponding to ± 4–8% muscle fiber strain) were close to that reported for mouse soleus muscle during locomotion. The experiments were performed at 32°C. The contributions of cross-bridge cycling and muscle activation to total metabolic energy expenditure were separated using blebbistatin. The mechanical work per contraction cycle decreased sharply during the first 10 cycles, emphasizing the importance of prolonged series of contractions. The mean ± SD fraction of metabolic energy required for activation was 0.37 ± 0.07 and 0.56 ± 0.17 for concentric and eccentric contractions, respectively (both 0.25 mm, 2 Hz). The mechanical efficiency during concentric contractions increased with contraction velocity from 0.12 ± 0.03 (0.25 mm 2 Hz) to 0.15 ± 0.03 (0.25 mm, 3 Hz) and 0.16 ± 0.02 (0.50 mm, 2 Hz) and was -0.22 ± 0.08 during eccentric contractions (0.25 mm, 2 Hz). The percentage of type I fibers correlated positively with mechanical efficiency during concentric contractions, but did not correlate with the fraction of metabolic energy required for activation.

## Introduction

Metabolic energy consumption is an important variable in whole body movement tasks. For tasks in which predominantly positive or negative muscle fiber mechanical work is performed, the relationship between mechanical behavior and metabolic energy expenditure is well established (Abbott et al., 1952; Woledge et al., 1985; Chavarren and Calbet, 1999; Linari et al., 2003). However, during periodic movements that occur in walking, metabolic energy expenditure is difficult to predict as the net external mechanical work done on the body is typically much smaller than the positive mechanical work delivered by the muscle fibers; the difference equalling the negative mechanical work done by the muscle fibers. Furthermore, the rate of change of the total mechanical energy of the body, within one movement cycle, is not one-to-one related to the sum of muscle fiber mechanical power, due to elastic energy storage and return in tendons and co-contraction of antagonist muscles. This further complicates estimation of the total positive muscle fiber mechanical work and metabolic energy expenditure during periodic movements. Musculoskeletal modeling in combination with optimization (e.g., Umberger, 2010; Miller et al., 2012; Markowitz and Herr, 2016) has the potential to contribute to our understanding of the mechanics and energetics of locomotion-related periodic movements. In order to realize this potential, musculoskeletal models must be developed that accurately predict both mechanical and metabolic behavior. The latter requires detailed knowledge of the separate contribution to metabolic energy consumption of all relevant processes which occur during muscular contraction.

There are two key processes which require energy during muscle contraction: cross-bridge cycling and the activation/relaxation process (Gordon et al., 2000), the latter being dominated by metabolically costly active calcium ion transport. Similar to previous studies, we define muscle’s baseline-subtracted mechanical efficiency as the net mechanical work done divided by the total metabolic energy expenditure minus the metabolic energy expenditure during rest. At the single muscle level, both baseline-subtracted mechanical efficiency (for reviews, see: Woledge et al., 1985; Smith et al., 2005) and the relative contribution of the activation process to metabolic energy consumption (for reviews, see Barclay et al., 2007; Loiselle et al., 2016) have been extensively studied in poikilothermic animals, and to a lesser extent in mammals, the latter being more relevant to our understanding of human locomotion. In the study of mammalian muscle’s mechanical efficiency at the single muscle level, a distinction is usually made between the “initial” efficiency and “net” efficiency. The first equals the net mechanical work divided by the baseline-subtracted metabolic energy expenditure during and immediately after contraction (due to net PCr splitting), whereas in the second the oxidative recovery after contraction is also accounted for in the metabolic energy expenditure (Smith et al., 2005). It has been reported that during concentric contractions initial and net efficiency differ by a factor ∼2; the maximal initial efficiency of isolated mammalian muscle is usually reported to be ∼0.30, whereas the maximal net efficiency equals ∼0.15 (Barclay and Weber, 2004; Smith et al., 2005; Barclay et al., 2010). For the purpose of comparison to the level of the whole organism, net efficiency is the more relevant measure, as this is what can be measured at the whole organism level. Unless stated otherwise, in this paper the term “mechanical efficiency” will refer to baseline-subtracted mechanical efficiency (i.e., “net” efficiency in Smith et al., 2005).

Under the assumption that the energy required to phosphorylate creatine is independent of the process for which ATP is used (i.e., the efficiency of the recovery process is invariant), the fraction of metabolic energy required for muscle (de)activation is the same when quantified using either initial or net metabolic energy expenditure. Using a variety of methods, the latter fraction has been estimated to be between 0.30 and 0.40 during short duration (<1 s) isometric contractions (Barclay et al., 2007; Loiselle et al., 2016). These results show that the metabolic energetic expenditure associated with (de)activation of muscle may be substantial. However, it is currently unclear to what extent these fractions are representative for muscles that undergo prolonged dynamical contractions, which occur during locomotion. Lewis and Barclay (2014) have quantified both the contribution of the activation process to metabolic energy consumption, and the mechanical efficiency in mammalian skeletal muscle (mouse soleus and extensor digitorum longus) during dynamic contractions (isovelocity shortening with 1.3^∗^*L*_0_/s; *L*_0_ was muscle optimum length). They observed that both the relative contribution of the overall (de)activation to metabolic energy expenditure, and the mechanical efficiency are dependent on the activation level, with a maximum of 0.30 and 0.14, respectively, for mouse soleus muscle during concentric contractions at high activation. Importantly, Lewis and Barclay (2014) did take into account the oxidative recovery, although the muscles might not have reached a metabolic steady state during the 10 contractions that were imposed. Moreover, in the context of locomotion, eccentric contractions are important. It is currently an open question whether the absolute amount of metabolic energy required for activation is equal for concentric and eccentric contractions.

The experimental data that is currently available to calibrate models of muscle energy expenditure typically concerns not more than a few contractions per trial (cf., Umberger et al., 2003; Umberger and Martin, 2007; Tsianos et al., 2012). This is problematic, as models of muscle energy expenditure are typically employed to predict metabolic energy expenditure of the whole body during prolonged, periodic movements. The aim of the present study was to quantify both the contribution of the activation process to total metabolic energy expenditure, and the mechanical efficiency, during prolonged contractions which are relevant for locomotion. To this end, we subjected small fiber bundles from mouse soleus muscle to sinusoidal contractions, with stimulation of bundles during either the concentric or the eccentric phase, while measuring force at the tendon. Contractions were imposed for 2 min and the experiments were carried out at 32°C. Metabolic energy expenditure was estimated from measurement of the bundles’ oxygen consumption. The contributions of cross-bridge cycling and activation to total metabolic energy expenditure were separated by application of blebbistatin (Straight et al., 2003). Finally, the relation between fiber type composition and energetics of muscle fiber contraction was explored.

## Materials and Methods

### Ethics Statement

All experimental procedures were performed in accordance with the regulations of the VU University Animal Experimentation Ethical committee (protocol number: DEC FBW1101) and were in accordance with Dutch law. Care was taken to minimize pain and distress of the animals at all times. Animals were housed at the VU University animal experiment center under a 12 h on/off light regime, and had access to chow and water *ad libitum*.

### Animals and Surgery

C57BL/6J mice (*N* = 9, five males, mass 27.2 ± 2.5 g) were placed on a heating patch (34°C) and anesthetized by inhalation of a gas mixture consisting of equal volumes of room air and oxygen, and 1.5 vol. % isoflurane. During surgery a gas mixture of 0.5 vol. % isoflurane was inhaled by the animal. Immediately after anaesthetization the hind limbs were shaved and a small patch of skin was removed. The temperature of the m. soleus was recorded using a fine tipped thermometer, which was placed underneath the muscle. The recorded temperature was 33.2 ± 0.9°C. Left and right m. soleus were dissected, including distal and proximal tendons. Muscles were immediately thereafter transferred to chilled Tyrode solution (in mM, 120 NaCl, 5 KCl, 1 CaCl_2_, 27 NaHCO_3_, 2 Na_2_HPO_4_, and 1.2 MgSO_4_) equilibrated with 95% O_2_/5% CO_2_. After surgery the animal was killed by a cardiac injection of pentobarbital sodium (0.5 mL, 20% Euthasol; AST Farma, Oudewater, Netherlands).

One of the muscles was placed in a dissection chamber perfused with Tyrode solution, and a small bundle of ∼70 ± 20 fibers was dissected free from the middle part of the muscle, under a dissection microscope fitted with darkfield illumination. Platinum rings were securely tied to the tendons of the muscle fiber bundle (hereafter referred to as bundle) close to the musculotendinous junction, using nylon thread 10 μm in diameter. Hereafter, bundle length and the largest and smallest diameters at three locations along its length were measured using an ocular scale. The latter measurements were used to estimate the bundle’s volume under the assumption its shape is that of an elliptical cylinder.

### Experimental Setup

The setup is described in detail by van der Laarse et al. (1989) and Wong et al. (2010). In short, after preparation, the bundle was transferred to a 381 mm^3^, glass, jacketed chamber, which was filled with Tyrode solution to which glucose (10 mM) was added. The water inside the water jacket was maintained at 32°C, close to the recorded *in vivo* muscle temperature. The proximal end of the bundle was attached to the bottom of the chamber. The distal end was attached to a tungsten wire, which left the chamber at the top via a thin capillary and was attached to a servomotor via a force transducer (AE801 Sensonor, Horten Norway). The bundle resting length was manually adjusted using a micromanipulator. A custom made, polarographic oxygen sensor protruded into the chamber through a tightly fitting opening. The resolution of the oxygen measurement is sufficient to measure oxygen consumption of a single frog muscle fiber at 20 degrees Celsius (Elzinga and van der Laarse, 1988). Through a separate opening, fresh Tyrode solution could be pumped into the chamber. The solution inside the chamber was circulated by a stainless-steel stirrer, which was driven by a magnetic field generated at the bottom of the chamber. The poles of an isolated stimulus generator were attached to stimulus leads placed at the bottom and at the top of the chamber and which were in contact with the Tyrode solution inside. The stimulus isolator generated alternating pulses of 0.4 ms width. In a separate experiment, without a bundle present in the chamber, it was verified that both stimulation and movement of the tungsten wire had no effect on the oxygen signal. Signals from the force transducer, motor position sensor, oxygen electrode and stimulus leads were A/D converted (12 bits) and sampled on a computer at 2 kHz. Prior to each experiment, the force transducer and the oxygen electrode were carefully calibrated. The oxygen sensor was calibrated by measuring the output while the electrode was suspended in 32°C Tyrode solution equilibrated with gas containing either 95% O_2_/5% CO_2_, room air (20.94% O_2_) or 100% N_2_.

### Experimental Protocols

Bundle length and stimulus amplitude leading to maximal twitch force were determined iteratively. For the remainder of the experiment, stimulus amplitude was set 10% above the threshold eliciting maximal twitch force. Next, 100 Hz tetanic stimulation (20 pulses per contraction) was applied at systematically varied lengths to characterize the force-length curve. The resting length of the bundle, hereafter referred to as bundle rest length, was set 0.5 mm below the length at which maximum force was recorded, in order to avoid damage associated with eccentric contraction at or above muscle fiber optimum length. Each measurement in which oxygen consumption was measured, hereafter referred to as a trial, started with stopping the supply of fresh Tyrode solution. The bundle was then subjected to sinusoidal length changes (2 or 3 Hz at 0.25 mm amplitude; or 2 Hz at 0.50 mm amplitude) and was stimulated (five pulses delivered at 100 Hz) during each cycle for 2 min (see the top inset in Figure 3A). For the 2 Hz, 0.25 mm movement amplitude condition, stimulus phase was set either at 90 or at 270 degrees relative to the beginning of the sine wave. The phase of the stimulation was chosen to result in a purely concentric (90 degrees) and a purely eccentric (270 degrees) condition. For all other conditions, stimulus phase was set at 90 degrees during all trials. Resting oxygen consumption was measured during 2 min prior, and 4 min after the stimulation period. After each measurement, fresh Tyrode solution was pumped through the chamber at a rate of 30 mL/h for ∼10 min. During this time, the bundle was subjected to various short duration (<1 s stimulation) dynamic contractions, which after the first trial included a set of contractions to characterize the tetanic stimulation frequency – isometric force relation. The maximum duration of one stimulation period was 200 ms. This stimulation duration was used for each contraction of the stimulation frequency – isometric force relation. As a consequence, during the lower stimulation frequency contractions, less pulses were administered than during the high stimulation frequency contractions, and force did not reach a plateau at the end of the contractions in which fewer pulses were delivered. Between trials, the short contractions always included an isometric contraction at the muscle rest length, with 20 pulses of tetanic stimulation delivered at 100 Hz. The force reached in this trial was compared to the initial plateau level of the force-length curve, in order to assess the stability of the bundle. Trials for which the prior isometric contraction yielded a maximum force <70% of the initial maximal force were excluded from analysis. Each trial required ∼25 min to complete, including the recovery period. Since the oxygen measurement was the most critical, the quality of the oxygen signal was assessed online and trials that were deemed invalid were repeated. An example of an oxygen signal containing an artifact is shown in Appendix Figure 1A. The success rate of the oxygen measurements was ∼70%. After all conditions were completed, the flow of fresh Tyrode was stopped and blebbistatin in Tyrode solution was injected into the chamber to a final concentration of 15 μM (Barclay, 2012). Blebbistatin was kept in the dark prior to injection into the chamber (which was shielded from light). Blebbistatin blocks myosin II in an actin detached state, thus disabling the cross-bridges. Processes other than the cross-bridge cycle, most importantly calcium transients, are unaffected by blebbistatin (Kovács et al., 2004; Farman et al., 2008). After admission of blebbistatin, we evaluated tetanic force every 30 s. Once tetanic force was no longer discernible, i.e., smaller than the resolution of our measurement system (<0.01 mN), the blebbistatin was flushed out of the chamber with fresh Tyrode. It usually took around 15 min for the force to completely vanish. After this procedure, the 2 Hz, 0.25 mm movement amplitude concentric and eccentric conditions were repeated. The order of concentric and eccentric trials was randomized both in the pre- and post-blebbistatin conditions.

After all trials were completed, the chamber was opened, and the total bundle length and the muscle fiber length at rest length were measured under a microscope using an ocular scale. Total bundle length was taken to be the distance between the clips, with the bundle at its resting length (i.e., the mean of the imposed sine waves). The muscle fiber length was taken to be the distance between the aponeuroses, in the direction of the muscle fiber, measured at the same bundle rest length. Note that ideally, the imposed length changes during the sine waves would have been bundle specific, based on the bundle’s optimum length for generating tetanic force. However, because the chamber assembly was a critical procedure, this was only done once (and once dissembled at the end). Consequently, we did not measure the absolute bundle length corresponding to the optimum bundle length prior to imposing the sine waves. This is the reason for imposing absolute, rather than relative length changes to the bundles. Note that the fiber length at rest also serves as our (best) estimate of optimum fiber length, as we had no way of measuring the fiber length during contractions. The imposed contraction velocities on the muscle fiber level are subject to a similar uncertainty. It is expected that the length changes imposed on the bundle are related, but not equal to the fiber length changes, as some of the length change will be taken up by the aponeuroses. However, at peak force, the fiber’s contraction velocity will be close to the imposed contraction velocity, because the derivative of the force and, consequently, aponeuroses’ length will be zero. As both length and velocity are continuously changing during these sinusoids, we reported the bundle’s length and contraction velocity at peak force as the best estimate of the overall bundle length and fiber contraction velocity, during contractions. After the length measurements inside the chamber, the bundle was transferred back to the dissection chamber and volume was again determined. The cross-sectional area of the bundle was taken as its volume divided by its muscle fiber length measured while suspended in the chamber at rest length. Finally, the bundle was embedded in Tyrode solution containing 15% gelatine (w/v) and stored in liquid nitrogen for subsequent histochemical analysis. The entire experimental procedure required ∼11 h.

### Histochemistry

Fiber type distribution and succinate dehydrogenase (SDH) activity were determined using histochemistry (van der Laarse et al., 1989; Bekedam et al., 2003). In short, 10 μm thick sections were cut using a cryostat at -20°C. SDH activity was determined immediately after sectioning, whereas sections for myosin immunohistochemistry were stored at -80°C for further analysis. Sections were stained for type I, IIA, IIB, and IIX myosin heavy chain as previously described (Ballak et al., 2014). For SDH activity, the sections were incubated in the dark for 10 min at 32°C in a medium consisting of 0.4 mM tetranitroblue tetrazolium (Sigma, St. Louis, MO, United States), 75 mM sodium succinate, 5 mM sodium azide, and 37.5 mM sodium phosphate buffer, pH 7.6. Images were made with 10× magnification objective using a Leica DMRB microscope (Wetzlar, Germany) with calibrated gray filters and a CCD camera (Sony XC77CE, Towada, Japan) connected to a LG-3 frame grabber (Scion, Frederick, MD, United States). SDH activity was quantified in individual fibers as the average absorbance of light at a wavelength of 660 nm (A660) (van der Laarse et al., 1989). Subsequently, for each bundle the average absorbance was taken as the average cross-sectional area weighted absorbance of the fibers. For each bundle the average absorbance was normalized by the section thickness and the incubation time (A660_norm_).

### Data Analysis

Aside from leakage through the capillary and o-rings, both the oxygen electrode itself and the stirrer reduce oxygen, without muscle fibers being present inside the chamber. Moreover, it was not feasible to work sterile, so there was an unknown contribution of micro-organisms to the measured oxygen consumption. The rate of oxygen loss from the chamber thus represents the sum of the bundle consumption and of other processes, not under experimental control. Net oxygen consumption was determined by subtracting for each trial separately the resting, baseline (chamber + bundle) oxygen consumption specific to that trial. The baseline (see Figure 3C) was constructed as a 2nd order polynomial fit on the oxygen signal (van der Laarse et al., 1989), which was defined by the following three conditions: (i) The slope at 60 s prior to stimulation and (ii) the slope 60 s prior to the end of the measurement was equal to the slope of the best fit straight line to, respectively, the first and last 120 s of the trial; and (iii) the baseline passes through the mean over the last 120 s of the trial, at 60 s prior to the end of the trial. Prior to constructing the baseline, the oxygen signal was low-pass filtered (zero lag, bidirectional Butterworth filter with 25 Hz cut-off frequency). Note that net oxygen consumption during activation may be influenced by changes in basal metabolism occurring upon stimulation. Although we have no way of quantifying this, we do not expect basal metabolism to be influenced to a large extent by the stimulation. The temperature of the medium was tightly controlled, so heat produced by the contraction would not have accumulated. Moreover, if basal metabolism would have been altered in response to stimulation, it would influence both the pre- and post-blebbistatin trials, such that the energy required for activation is still adequately measured. Finally, basal metabolism contributes only a small part to the total metabolism during active contractions. Thus, although we have no hard evidence for this, we expect that increased basal metabolism contributed at best a small part of the total change in metabolism observed during contractions. In addition to the online check performed during the experiment, the quality of oxygen signals was assessed after subtraction of the baseline and trials for which the oxygen signal contained artifacts were discarded. Altogether, the above method did not allow estimation of the bundle’s resting metabolic rate, but did yield a reliable estimate of the net oxygen consumption in response to the stimulation.

For *in vitro* preparations, an important prerequisite for steady state behavior is that the preparation is small enough to allow the required oxygen to reach all fibers through diffusion (Hill, 1928). To check for this condition, we calculated for each trial the largest possible, smallest diameter of the bundle that would still allow for steady state diffusive oxygen supply, using equation 3 from Barclay (2005), the mean oxygen consumption observed during the trial, and the lowest value of oxygen pressure observed during the trial. Comparison of this critical radius to the smallest diameter of each preparation indicated to what extent diffusive oxygen supply could be a limiting factor in the present experiments.

Net mechanical work exerted by/on the bundle was calculated from the force and length traces (e.g., Figure 3A,B) by trapezoidal integration of force with respect to length change. Metabolic energy expenditure was calculated from the baseline subtracted oxygen signal (see above), using the chamber volume (381 mm^3^), the solubility of oxygen in Tyrode solution at 32°C (1.084 nmol/mm^3^; Bartels et al., 1971) and a metabolic equivalent of oxygen of 473 kJ/mol (Carpenter, 1939). The latter value is based on the assumption of pure glucose oxidation. In a pilot experiment, we repeated the 0.25 mm, 2 Hz concentric condition several times, but did not add glucose to the Tyrode solution. In this experiment, the bundle showed an uncharacteristic decline in both oxygen consumption and mechanical power output; average mechanical power was 2.7, 2.4, and 1.4 W/kg for the 1st to 3rd trial, respectively. Upon replacing the Tyrode solution with Tyrode solution containing glucose, both the mechanical power output (1.6 W/kg) and the oxygen consumption showed a slight recovery at the 4th trial, and remained stable thereafter. This indicates that glucose addition to the Tyrode solution was essential for stability of the preparation.

### Statistics

All reported results are mean ± standard deviation of the sample from *N* = 9 mice, unless mentioned otherwise, in which case the results are from a subset of these mice. At the whole muscle level, male and female mice muscles differ in terms of muscle mass, maximum force and fiber type composition (Eason et al., 2000; Lionikas et al., 2003; Miller et al., 2013). However, for a muscle fiber bundle from young adult mice with given fiber type distribution, cross-sectional area and mass, we do not know of any reports of sexual dimorphism. Therefore, the results of male and female mice were pooled in the current study. All results in which a typical example is shown are of the same mouse. Repeated observations within bundles were averaged prior to averaging between bundles. Because of the small sample size, non-parametric statistics were used. Wilcoxon signed rank tests were performed to compare group means between conditions, for the mechanical efficiency and the fraction of metabolic energy required for activation. Spearman’s correlation was calculated between the percentage of type 1 fibers and both mechanical efficiency and fraction of metabolic energy required for activation. All statistical tests that were conducted are also mentioned below.

## Results

### General Muscle Fiber Bundle Characteristics

**FIGURE 1:**
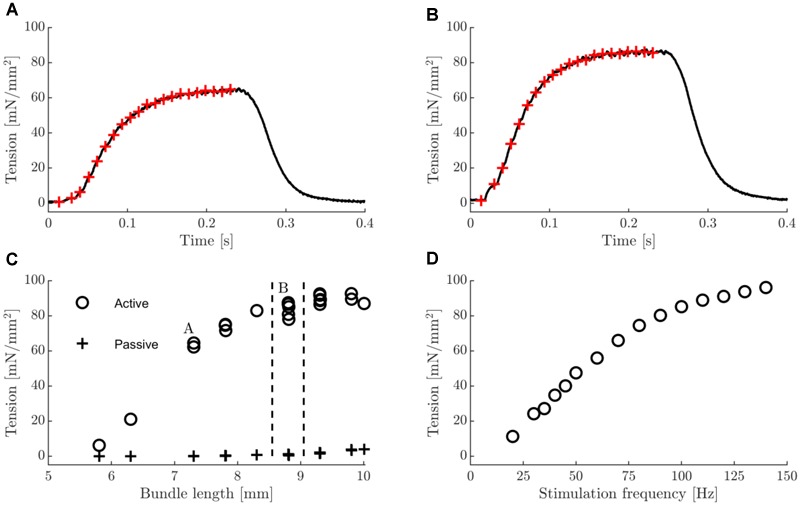
Typical examples of tetanic contractions, tension-length and tension-stimulation frequency relation. **(A)** Tetanic contraction on ascending part of tension length relationship. **(B)** Tetanic contraction close to optimal fiber bundle length. In panels **(A,B)**, red crosses indicate times at which stimulus pulses were applied (stimulation frequency was 100 Hz). **(C)** Active and passive tension-length relationship. In the active condition, each tetanus consisted of 20 stimulus pulses delivered at 100 Hz. Vertical dashed lines indicate the range of bundle lengths encountered during the work loops in 0.25 mm amplitude conditions. The datapoints labeled A and B correspond to the data in panels **(A,B)** of this figure. Multiple data points corresponding to a single length indicate repeated trials. **(D)** tetanic tension-stimulation frequency relationship. Each tetanus consisted of 20 stimulus pulses.

**FIGURE 2:**
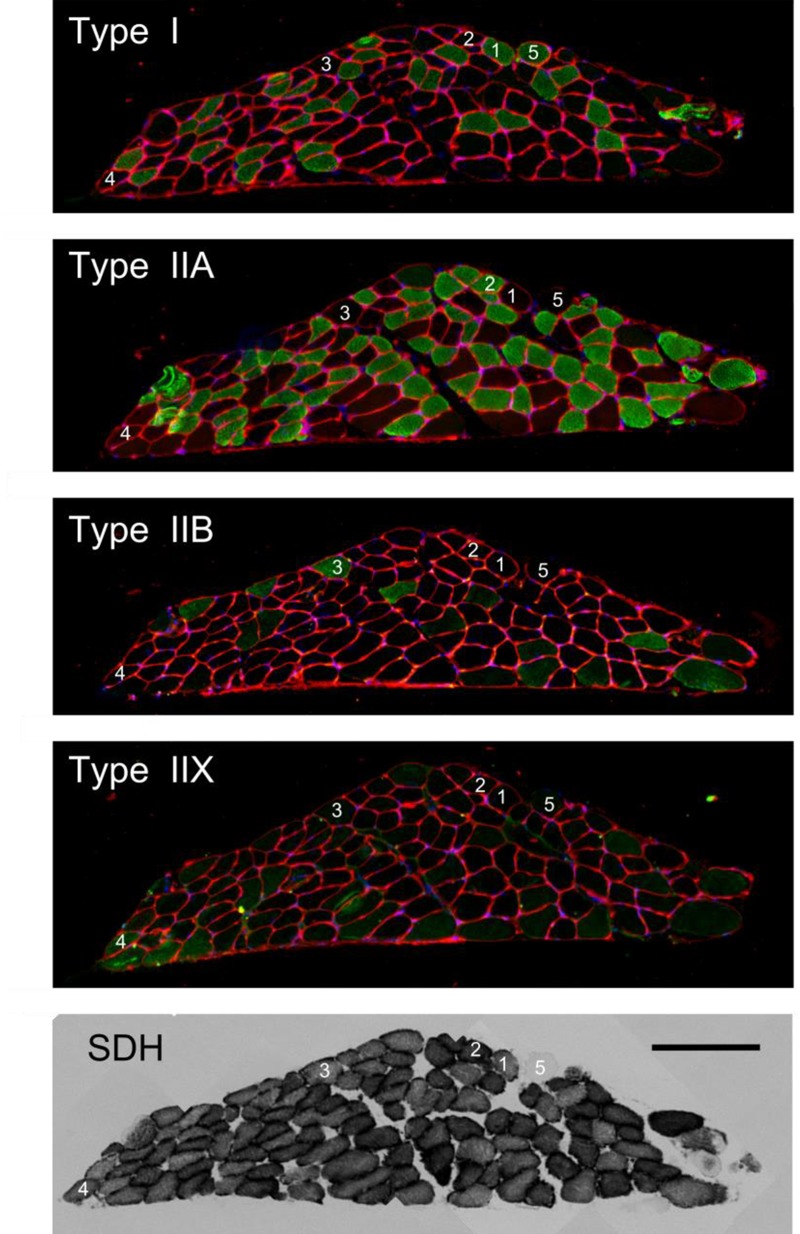
Typical example of histochemically stained cross-sections of a muscle fiber bundle. Type of staining is indicated within each image. The bottom right scale bar serves all panels and represents 200 μm. The numbered fibers 1–5 label Type I, IIA, IIB, IIX, and a fiber devoid of mitochondrial activity, respectively. Note that the fiber without mitochondrial activity (nr 5) is stained positive in the type I staining.

In Figure 1, typical examples of tetanic contractions (Figure 1A,B), a tension length relationship (Figure 1C), and a tenstion stimulus frequency relationship (Figure 1D) are shown. At all bundle lengths visited in the tension-length relationship, the force approached a steady state level at the end of the contraction (Figure 1A,B). Note that the length range that was imposed during the dynamic trials was at the lower end of the tension-length plateau, such that damage from eccentric contractions above optimum length was avoided. Further note that the tension – stimulation frequency curve is not yet saturated at 100 Hz stimulation frequency. However, we chose 100 Hz frequency as the stimulus frequency during all other trials, as we considered higher stimulation frequencies to be unrealistic during *in vivo* muscle operation. The group mean values of basic characteristics of the male and female mice were similar; animal mass was 28 g vs. 27 g; twitch tension was 11 mN/mm^2^ vs. 13 mN/mm^2^; twitch rise time was 17 ms vs. 18 ms; A660_norm_ was 6.2e-05 A660 ^∗^ s^-1^^∗^ μm^-1^ vs. 5.7e-05 A660 ^∗^ s^-1∗^ μm^-1^. In addition to the considerations put forward in the methods section, these results support pooling the data of male and female mice in all subsequent analysis. The general characteristics of the bundles are listed in Table 1. As expected, maximum force was positively correlated with cross-sectional area (Spearman’s rho = 0.78, *p* = 0.02). Volume determinations before and after the experiment were almost identical (mean difference -0.04 ± 0.14 mm^3^) and highly correlated (Spearman’s rho = 0.92, *p* = 0.001), indicating no fiber loss over the course of the experiment. As to be expected in a healthy preparation, bundle volume was correlated to both mechanical and metabolic power during the concentric 0.25 mm amplitude, 2 Hz movement frequency condition (Spearman’s rho = 0.67, *p* = 0.06 and Spearman’s rho = 0.73, *p* = 0.03, respectively). In Figure 2, a typical example of myosin heavy chain type and SDH activity is shown. Note that the number of cells in which mitochondrial activity is absent (recognizable by a round appearance and a lack of SDH activity, cf., example cell marked 5) is small, which was the case for all preparations. Further note that the bundle consisted primarily of low oxidative fibers (type I and IIA). The latter observation generalized to all bundles, as can be appreciated from Table 2, in which the fiber type percentages and the (normalized) average absorbance (SDH A660_norm_) are listed. Note that contrary to the shape assumed in the calculation of muscle volume, the cross-section of the fiber bundle did not resemble an ellipsoid, which was the case for most fiber bundles. This is most likely due to the embedding procedure, which may have altered the shape of fiber bundle.

**Table 1:** Mean ± SD of bundle characteristics (*N* = 9).

Mass (mg)	1.1 ± 0.4
Cross sectional area (mm^2^)	0.17 ± 0.04
Bundle rest length (i.e., mean of sinusoids) (mm)	8.1 ± 0.8
Fiber length at bundle rest length (mm)	5.8 ± 0.8
Bundle length at maximal isometric force (mm)	8.9 ± 0.8
Maximum isometric force (mN)	17.3 ± 3.8
Maximum isometric tension (mN/mm^2^)	101 ± 16
Peak twitch tension (mN/mm^2^)	12 ± 4
Twitch rise time (ms)	17 ± 2
Twitch relaxation time (ms)	50 ± 10

*Twitch contraction/relaxation time is the time between onset/relaxation of force and peak force, during a contraction elicited by a single excitation pulse.*

**Table 2:** Mean ± SD of bundle fiber type distribution, average SDH activity (A660_norm_).

% Type I fibers	37 ± 11
% Type I/IIA fibers	17 ± 6, *N* = 3
% Type IIA fibers	53 ± 13
% Type IIB fibers	6 ± 1, *N* = 2
% Type IIX fibers	7 ± 1, *N* = 2
SDH A660_norm_ (A660 ^∗^ s^-1^^∗^ μm^-1^)	5.9e-5 ± 7.1e-6

*N = 9 mice, unless indicated otherwise.*

### Net Mechanical Efficiency Increased With Contraction Velocity

**FIGURE 3:**
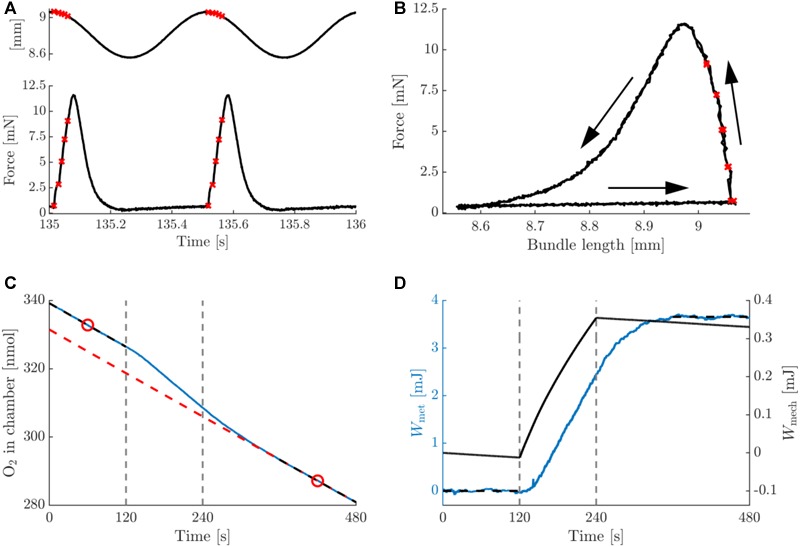
Summary data for a typical example of a concentric trial at a movement frequency of 2 Hz and amplitude 0.25 mm, pre-blebbistatin. **(A)** Bundle length (top inset) and force against time, relative to the onset of the trial. **(B)** Work loop depiction (force against bundle length) of the data in panel **(A)**, the two cycles are superimposed. Arrows indicate the progression of time. The red crosses in panels **(A,B)** indicate instances at which stimuli were delivered. **(C)** Total amount of oxygen present in the chamber (blue, solid line), and the baseline oxygen trace of the chamber + bundle resting consumption (red, dashed line), against time. The black, dashed line sections are linear fits to the first and last 2 min of the oxygen trace, and the red circles are the means of these fits. The baseline is a 2nd degree polynomial of which the slope at the time points given by the red circles is equal to the slope of the respective black dashed lines, and which passes through the rightmost red circle. Vertical dashed lines indicate the start and end of the stimulation period. **(D)** left (blue) axis: net (baseline subtracted) oxygen consumption of bundle expressed as caloric equivalent, against time. The net amount of energy liberated by the bundle during the trial was computed as the difference between the mean of the first and last 2 min of the trial (horizontal black, dashed lines). Right (black) axis: total mechanical work computed as the integral of force with respect to bundle length change, against time. The net mechanical work associated with the trial was computed as the difference between the values at the beginning and the end of the stimulation period (vertical, dashed lines). Note that the work calculated directly from panel **(B)** would yield a net negative value per cycle, as per convention the force from the muscle on the environment is depicted positive, whereas in the calculation of mechanical work it was taken to be negative.

**FIGURE 4:**
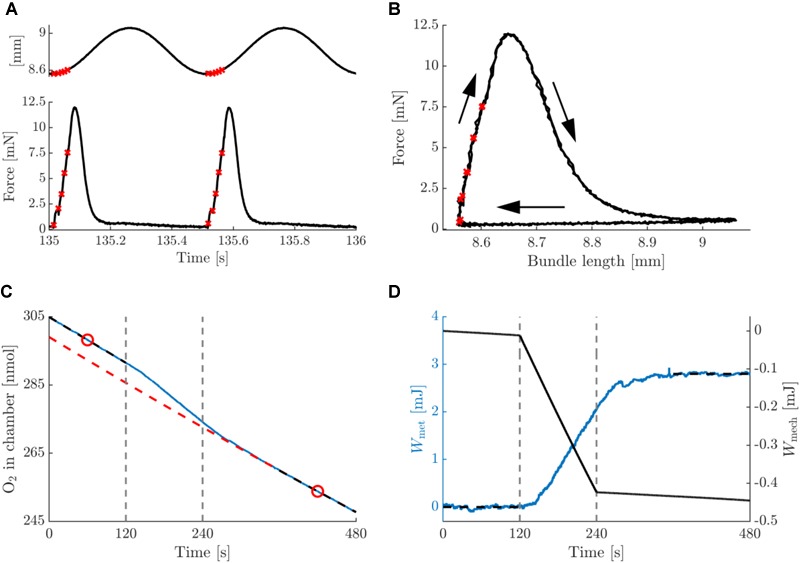
Summary data for a typical example of an eccentric contraction with amplitude 0.25 mm and movement frequency 2 Hz, pre-blebbistatin. Figure lay-out identical to that of Figure 3; see legend thereof.

In Figure 3, typical data on the mechanics and energetics of a pre-blebbistatin, concentric trial, at 2 Hz movement frequency and 0.25 mm movement amplitude are shown. In the passive phase (i.e., no stimulation), a small amount of mechanical energy was dissipated in the bundle (Figure 3D, right axis), and a non-negligible rate of change of oxygen content in the chamber was observed (see section “Materials and Methods”) (Figure 3C). The stimulation period resulted in active force during shortening only (Figure 3A), and hence the generation of net positive mechanical work per cycle (Figure 3B). The latter was accompanied by a substantial increase in the rate of oxygen consumption (Figure 3C). As can be appreciated from Figure 3D, left axis, the baseline subtraction resulted in a net metabolic energy expenditure of the bundle which was elevated during the stimulation and the recovery period. Mean metabolic power was estimated by dividing net metabolic energy consumption by the duration of the stimulus period (i.e., 120 s). During the stimulation period, the bundle’s mechanical power production gradually decreased, as can be seen from the change in slope of the mechanical work (Figure 3D). Because the bundles were thus not strictly in steady state, we chose to relate the net amount of metabolic energy expenditure in each trial to the corresponding net amount of mechanical work, as depicted in Figure 3D. The analog eccentric case is depicted in Figure 4. Note that for ease of comparison, the layout and the range on the y-axes are similar to those in Figure 3. As expected, during the pre-blebbistatin eccentric trial an amount of mechanical energy was dissipated that was higher than that generated during the pre-blebbistatin concentric trial, at the expense of less metabolic energy. These findings generalize to all animals; the mean mechanical and metabolic power during the pre-blebbistatin trials are listed in Table 3, first six rows. In the 0.25 mm, 2 Hz pre-blebbistatin condition metabolic power was lower for eccentric as compared to concentric contractions, whereas absolute mechanical power was higher in the eccentric condition. This resulted in a “higher” “eccentric mechanical efficiency” (-0.22 ± 0.08) as compared to concentric mechanical efficiency (0.12 ± 0.03). In the 2 Hz, 0.25 mm condition, the average contraction velocity at peak force was -2.4 ± 0.1 mm/s (-0.43 ± 0.06 fiber rest length/s) and 2.5 ± 0.2 mm/s (0.43 ± 0.05 fiber rest length/s) for concentric and eccentric contractions, respectively. In concentric trials, when increasing movement frequency to 3 Hz, while keeping the number of stimulus pulses per cycle equal, the average contraction velocity at peak force increased to -4.5 ± 0.1 mm/s (-0.77 ± 0.10 fiber rest length/s). For these trials, the mechanical power output increased more than metabolic power, resulting in a significant increase in concentric mechanical efficiency to 0.15 ± 0.03; signed rank = 0, *p* = 0.016, *N* = 7. Doubling movement amplitude to 0.5 mm in the 2 Hz condition, resulted in an average contraction velocity of -4.2 ± 0.2 mm/s (-0.70 ± 0.09 fiber rest length/s) and in an increase of mechanical efficiency to 0.16 ± 0.02; signed rank = 0, *p* = 0.031, *N* = 6. The mean mechanical efficiency for all trials is listed in Table 4, first three rows.

**Table 3:** Mean ± SD mechanical power and net metabolic power for all conditions, averaged across mice.

	Concentric contraction	Eccentric contraction	Difference of absolute values
Pmech (W/kg), 0.25 mm, 2 Hz, B0	2.7 ± 0.6	-3.1 ± 1.2	-0.4 ± 0.9
Pmech (W/kg), 0.25 mm, 3 Hz, B0	3.6 ± 1.5, *N* = 7	n.a.	n.a.
Pmech (W/kg), 0.50 mm, 2 Hz, B0	3.9 ± 1.0, *N* = 6	n.a.	n.a.
Pmet (W/kg), 0.25 mm, 2 Hz, B0	21.4 ± 3.2	15.0 ± 4.3	6.4 ± 5.1
Pmet (W/kg), 0.25 mm, 3 Hz, B0	23.4 ± 6.8, *N* = 7	n.a.	n.a.
Pmet (W/kg), 0.50 mm, 2 Hz, B0	23.4 ± 4.3, *N* = 6	n.a.	n.a.
Pmech (W/kg), 0.25 mm, 2 Hz, B1	-0.1 ± 0.1, *N* = 7	-0.1 ± 0.1, *N* = 6	-0.0 ± 0.0, *N* = 6
Pmet (W/kg), 0.25 mm, 2 Hz, B1	7.4 ± 1.6, *N* = 7	8.8 ± 2.8, *N* = 6	-1.3 ± 1.3, *N* = 6

*N = 9 mice, unless indicated otherwise. In all trials, length varied sinusoidally with amplitude 0.25 or 0.50 mm, and movement frequency was either 2 or 3 Hz. At both movement frequencies, five pulses (at 100 Hz) were delivered at either 90 (concentric) or 270 (eccentric) degrees of phase relative to the start of the sine wave, during each cycle. B0: pre-blebbistatin. B1: post-blebbistatin. The values reported in column 4 are the means and standard deviations of the paired differences between concentric and eccentric contraction. n.a., not available.*

**Table 4:** Mean ± SD mechanical efficiency and the fraction of metabolic power required for the activation process, for all conditions, averaged across mice.

	Concentric	Eccentric	Difference of absolute values
Efficiency, 0.25 mm, 2 Hz	0.12 ± 0.03	-0.22 ± 0.08	-0.10 ± 0.06
Efficiency, 0.25 mm, 3 Hz	0.15 ± 0.03, *N* = 7	n.a.	n.a.
Efficiency, 0.50 mm, 2 Hz	0.16 ± 0.02, *N* = 6	n.a.	n.a.
Activation fraction of Pmet, 0.25 mm, 2 Hz	0.37 ± 0.07, *N* = 7	0.56 ± 0.17, *N* = 6	-0.18 ± 0.12, *N* = 6

*N = 9 mice, unless indicated otherwise. In all trials, length varied sinusoidally with amplitude 0.25 or 0.50 mm, and movement frequency was either 2 or 3 Hz. At both movement frequencies, five pulses (at 100 Hz) were given at either 90 (concentric) or 270 (eccentric) degrees of phase relative to the start of the sine wave, during each cycle. The values reported in column 4 are the means and standard deviations of the paired differences between concentric and eccentric contraction. Values in this table were calculated from the raw data underlying Table 3. n.a., not available.*

### Metabolic Energy Required for Activation Was Substantial

**FIGURE 5:**
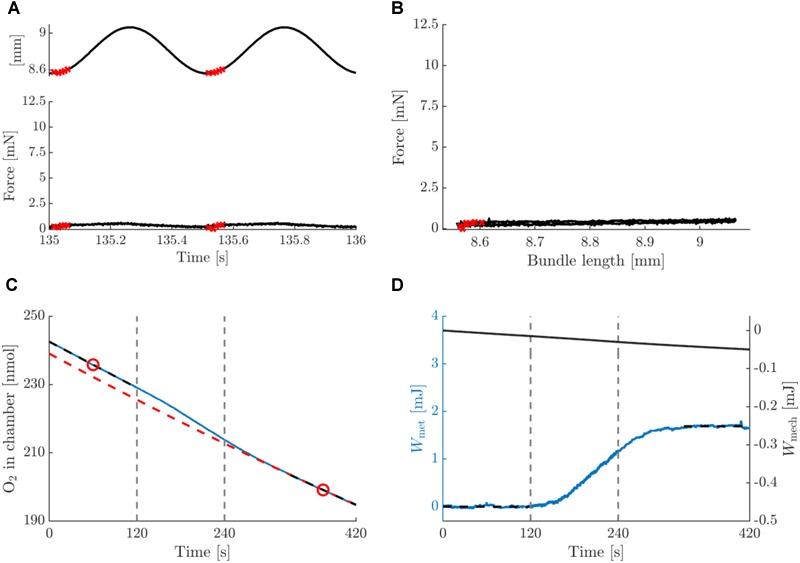
Summary data for a typical example of a concentric contraction with amplitude 0.25 mm and movement frequency 2 Hz, post-blebbistatin. Figure lay-out identical to that of Figure 3; see legend thereof.

**FIGURE 6:**
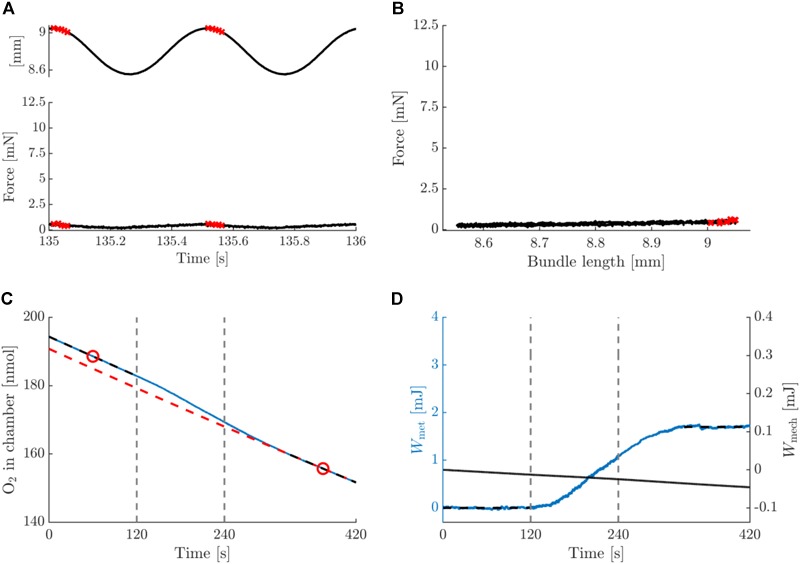
Summary data for a typical example of an eccentric contraction with amplitude 0.25 mm and movement frequency 2 Hz, post-blebbistatin. Figure lay-out identical to that of Figure 3; see legend thereof.

Figure 5, 6 depict a typical example of a post-blebbistatin concentric and eccentric trial, respectively, both at 2 Hz movement frequency and 0.25 mm amplitude. For ease of comparison, Figure 5, 6 have again the same layout and the same range on the ordinate as Figure 3, 4. As expected, the bundles did not generate any force upon stimulation during post-blebbistatin trials, irrespective of contraction condition (Figure 5A,B, 6A,B), showing that the cross-bridges were no longer functional. Furthermore, although no active force was generated, a substantial amount of metabolic energy was expended by the bundle during the stimulation period, which we ascribe to the activation process (Figure 5C,D, 6C,D). The absolute metabolic power required for muscle activation did not differ between concentric (7.4 ± 1.6 W/kg) and eccentric (8.8 ± 2.8 W/kg) contractions; signed rank = 2, *p* = 0.094, *N* = 6 (Table 3, last row). As the total amount of metabolic energy expenditure was smaller for eccentric compared to concentric contractions, the fraction of the total metabolic energy expenditure required for activation was significantly larger for eccentric (0.57 ± 0.17) than for concentric contractions (0.37 ± 0.07), signed rank = 0, *p* = 0.031, *N* = 6 (Table 4, last row).

### Net Mechanical Efficiency for Concentric Contractions Was Positively Related to the Percentage of Type I Fibers

**FIGURE 7:**
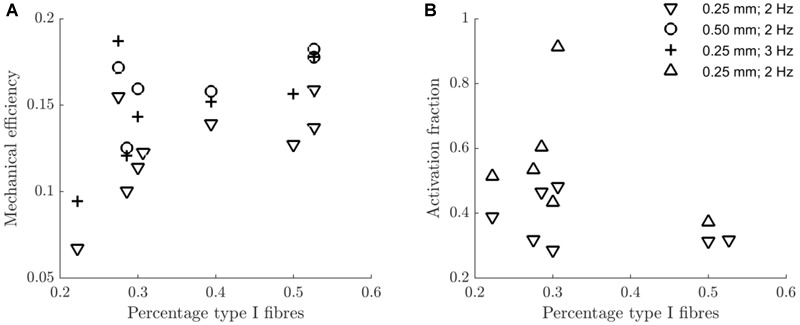
Scatterplot of the mechanical efficiency **(A)** and the fraction of metabolic energy spent on the activation process **(B)** against the percentage of type I fibers, for each contraction condition. The legend in panel **(B)** indicates the contraction conditions (amplitude and movement frequency), and serves both panels. The downward pointing triangles refer to concentric contraction and the upward pointing triangles refer to eccentric contraction. For each condition and variable, the Spearman correlation with the percentage of type I fibers is listed in Table 5.

In Figure 7 the mechanical efficiency (Figure 7A) and the fraction of metabolic energy required for activation (Figure 7B) as a function of the percentage of type I fibers are depicted, for each condition. In Table 5, the corresponding Spearman’s correlation coefficients are listed. For mechanical efficiency, there was a likely effect of fiber type. Judged over all concentric conditions, mechanical efficiency was positively correlated with the percentage of type I fibers, as expected for a relatively low contraction velocity task. There was no correlation between the fraction of metabolic energy required for activation and fiber type, irrespective of contraction condition. Unfortunately, we cannot make a definitive statement regarding this null-finding, as it may just reflect a limitation in power. Adding to this, the measure on the *y*-axis is a division between two noisy signals, resulting in the large spread of the data around 30% type I fibers, which may have obscured any relationship, if present.

**Table 5:** Spearman’s correlation coefficients and *p*-values of associations between the fraction of metabolic energy spent on activation/the mechanical efficiency, and the percentage of type 1 fibers.

Variable/condition	Spearman’s rho	*p*-value	Number of observations
Activation fraction, concentric	-0.43	0.354	7
Activation fraction, eccentric	-0.37	0.497	6
Efficiency, *a* = 0.25 mm; *f* = 2 Hz; concentric	0.78	0.017	9
Efficiency, *a* = 0.25 mm; *f* = 3 Hz; concentric	0.64	0.139	7
Efficiency, *a* = 0.50 mm; *f* = 2 Hz; concentric	0.66	0.175	6

### Within Trials, Mechanical Power Declined Over Time

**FIGURE 8:**
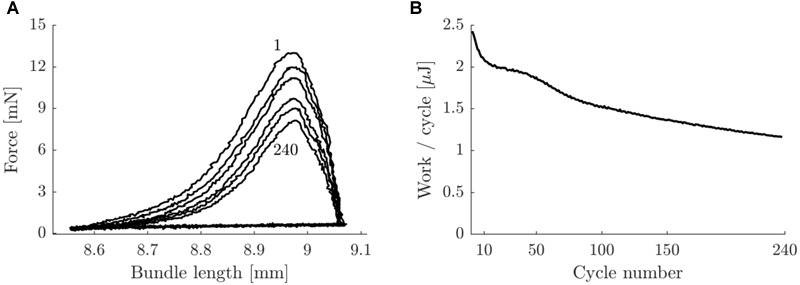
Typical example of work loop shape and work per cycle as a function of cycle number for a concentric trial with movement amplitude 0.25 mm and 2 Hz movement frequency, pre-blebbistatin. **(A)** Bundle force against length, for cycle numbers 1, 10, 50, 100, 150, and 240 (top to bottom curves). **(B)** Net mechanical work per cycle as a function of cycle number. Ticks on the *x*-axis correspond with lines in panel **(A)** (the tick at cycle number 1 was left out for readability). The data in this figure corresponds to the data in Figure 3.

In Figure 8 a typical example of the work loop shape and the mechanical work output per cycle as a function of cycle number (as counted from the start of the stimulation period), is shown. Note that the mechanical work output per cycle continuously decreases during the trial. This decrease was most prominent during the first 10 cycles of the stimulation period. Averaged over all concentric pre-blebbistatin trials, the average mechanical power output during the first and last 10 s of the stimulation period was 3.4 ± 1.0 W/kg and 2.3 ± 0.7 W/kg, respectively. For eccentric pre-blebbistatin trials the average mechanical power output during the first and last 10 s of the stimulation period was -3.5 ± 1.3 W/kg and -3.0 ± 1.2 W/kg, respectively.

### Preparations Remained Stable Over Consecutive Trials

Averaged over trials and animals (SD referring to variation between animals), the theoretical maximum value of the smallest radius of the bundle that allowed for diffusive oxygen supply under the given conditions was 0.38 ± 0.03 mm. The actual smallest radius of the bundles was 0.19 ± 0.03 mm. For none of the trials, the smallest radius exceeded the theoretical maximum value at which the oxygen pressure at the bundle’s core approaches zero. This observation renders it unlikely that diffusive oxygen supply was a limiting factor in the present experiments. In Table 6, the mechanical energy dissipated during the passive parts of the trial, and the metabolic energy consumed during the initial passive part of each trial are given. Note that the group average for passive mechanical energy dissipated did not change between pre- and post-blebbistatin conditions, and did not change between the initial and final passive part of each trial. Further note that averaged metabolic power (chamber + bundle) during the initial passive part of each trial did not change between pre- and post-blebbistatin conditions. The latter observations indicate that the bundles remained stable during the experiment.

**Table 6:** Mean ± SD average mechanical and metabolic power during the passive work loops.

	Pre-blebbistatin	Post-blebbistatin	Difference
Pmech, first 2 min (W/kg)	-0.26 ± 0.22	-0.17 ± 0.14	-0.09 ± 0.23
Pmech, last 4 min (W/kg)	-0.26 ± 0.20	-0.17 ± 0.13	-0.08 ± 0.21
Pmet, first 2 min (W/kg)	41.4 ± 11.9	44.2 ± 15.8	-2.7 ± 6.0

*Data were first averaged over all trials within each experiment, and then the means were taken across experimental days, SD referring to the latter. *N* = 9 for all data shown. For mechanical power (Pmech) every first 2 min and every last 4 min of each trial were considered. For the metabolic power (Pmet) the data shown here has been derived from the chamber + bundle oxygen consumption during the first 2 min of each trial. The values reported in column 4 are means and standard deviations of the paired differences between the trials prior and after admission of blebbistatin.*

## Discussion

The aim of this study was to quantify the contribution of muscle activation to total metabolic energy expenditure, and the mechanical efficiency, during contractions relevant for locomotion. To this end we measured oxygen consumption and mechanical power output during stretch-shortening cycles of small bundles of mouse muscle fibers, both before and after disabling the cross-bridge cycling, by use of blebbistatin. The main result was that the relative contribution of the activation process to total metabolic energy expenditure was substantial: 0.37 and 0.56 during concentric and eccentric contractions, respectively. In our view, it is understandable why a large amount of energy is expended on (de)activation. The main contributor to the metabolism associated with activation is believed to be the sarcoplasmatic calcium pump, which pumps calcium back into the sarcoplasmic reticulum in order to stop the contraction (Barclay, 2012). Rapid muscle relaxation is of critical importance in many motor tasks (e.g., generating positive work in a cyclic motion), and therefore may have been selected for in evolutionary history. Taking this into consideration, we consider it quite feasible to expend a substantial amount of energy to accommodate this feature. Whereas the relative contribution of activation energy to total metabolism depended on contraction condition, the mass normalized metabolic energy required for muscle activation did not differ between concentric (7.4 ± 1.6 W/kg) and eccentric (8.8 ± 2.8 W/kg) contractions. The absolute average mechanical power output was smaller during concentric than during eccentric contractions, and the average metabolic power was larger during concentric than during eccentric contractions. This resulted in a lower absolute mechanical efficiency during concentric contractions (0.12–0.16 for the conditions measured here) than during eccentric contractions (-0.22). A large part of the variation in mechanical efficiency was explained by the between-bundle variation in percentage of type 1 fibers. No significant correlation was found between the percentage of type 1 fibers and the fraction of metabolic energy required for the activation process.

### Validity of Results

The maximal specific tension reported in the current study (mean 101 mN/mm^2^) was on the low end of what is usually reported for mouse soleus muscle fiber bundles, i.e., ∼100–180 mN/mm^2^ for fiber bundles 3–5 mg (Lichtwark and Barclay, 2012; Lewis and Barclay, 2014). Moreover, the maximal specific tension reported here is lower than that reported for single mouse fibers (∼375 mN/mm^2^ Lannergren and Westerblad, 1987). In the sections stained for SDH-activity, only a very small proportion of cells was devoid of mitochondrial activity (appeared as blank, see fiber labeled 5 in Figure 2, bottom image). It is therefore not likely that the difference between the maximal tension measured in single fibers and in fiber bundles can be explained by inactive cells contributing substantially to bundle volume, but not to force output. With regard to the validity of our tension measurement we note that maximum force and cross-sectional area were well correlated and that the force transducer was carefully calibrated prior to each measurement day. Moreover, volume determinations before and after the experiment were almost identical and highly correlated, and bundle volume was highly correlated to both mechanical and metabolic power. We therefore consider our tension measurement to be valid. Note that for the calculation of cross-sectional area, the denominator is often taken to be total muscle length, rather than muscle fiber length. Taking total bundle length as the denominator in the current study would result in a muscle tension of 139 mN/mm^2^. An alternative explanation for the low maximum tension observed here, is that not all fibers were recruited during the supramaximal stimulation used in this study. This is because fibers at the core of the bundle may have been electrically isolated from the Tyrode solution and hence would not be excited. It was not possible to further increase the stimulus amplitude due to the risk of electrolysis in the medium, which would interfere with the oxygen measurement. If not all fibers were recruited, it could also be that a subset of fibers was recruited from a particular fiber type. The relatively slow twitch rise time (mean 17 ms) reported here suggests that mostly slow, type I fibers were recruited (cf Lannergren and Westerblad, 1987). However, we did not find a relation between the percentage of type I fibers and the maximum tetanic force. Note that if a substantial portion of the fibers was not recruited, all quantities that were normalized for mass or cross-sectional area would be underestimated. However, and importantly, the fraction of fibers that is recruited has no bearing on the mechanical efficiency and the relative contribution of the activation process reported here, since resting metabolism was subtracted in all trials.

In Figure 3, 4 peak force was similar in the concentric and eccentric contractions. A possible explanation for this is that during concentric contractions the bundle was stimulated at the top of the sine wave, whereas during eccentric contractions, the bundle was stimulated at the bottom of the same sine wave. To estimate the effect of this difference we compared the bundle length at which peak force occurred. This was at 1.02 ± 0.00 and 0.98 ± 0.00 bundle rest length, for concentric and eccentric contractions, respectively. Based on these numbers we do not expect that the force length relationship had a large influence on the difference between concentric and eccentric contractions. Overall, similar to mechanical work, peak force was 15% higher in eccentric compared to concentric contractions. Considering the relatively low contraction velocities imposed in this study, we consider this not an unexpected difference.

The same concentration of blebbistatin was applied to all bundles. The minimal concentration at which blebbistatin is effective is likely to depend on the muscle volume, and may also depend on fiber type. However, for us the critical test of blebbistatin’s efficacy was to observe that all force vanished both during the isometric test contractions and during the subsequent workloops. As a consequence of the irreversibility of the effect of blebbistatin, the measurements of oxygen consumption pre- and post-blebbistatin could not be counterbalanced. It is therefore important to assert that the bundles remained stable between trials, throughout the experiment. Stability was assured by the requirement on maximal isometric force to remain above 70% of its initial value when measured in between trials. Note that the average mechanical power output and force during passive work loops was small, and did not differ between the pre- and post-blebbistatin trials (Table 6). In addition to the small number of inactive cells observed in the sections stained for SDH activity, the latter similarity in passive mechanical power indicates that no fibers entered a state of rigor during the experiment, as this would have resulted in an increase in the passive force. In sum, we found no indication that fibers died during the experiment. Finally, based on calculation of the maximal diffusion radius (Barclay, 2005), we showed that in each trial oxygen pressure was sufficient to allow for the oxygen requirement of all fibers through diffusion: oxygen supply was not a limiting factor in the present experiment.

### Comparison to the Literature

The values reported in this study for the fraction of metabolic energy required for activation are close to previously reported values for prolonged concentric and isometric contractions based on heat measurements (Lewis and Barclay, 2014). As expected, we observed a “higher” eccentric mechanical efficiency compared to concentric mechanical efficiency. A generally accepted explanation for this observation is that cross-bridges can detach mechanically during eccentric contraction, at no energetic cost, whereas energy in the form of ATP splitting must be liberated to complete the cross-bridge cycle during concentric contraction. In addition to the latter mechanism, it is conceivable that part of the difference between eccentric and concentric mechanical efficiency is due to a difference in metabolic energy required for activation. However, we did not find evidence for such a difference here, as the average metabolic power required for activation did not differ statistically between concentric and eccentric contractions. The latter suggests that the energy required for activation is independent of contraction type. Our interpretation of this finding is that the workings of calcium pumps are not influenced by contraction mode.

The values for mechanical efficiency during concentric contraction reported in the present study are similar to those reported in other studies in which multiple contractions were measured and recovery heat was taken into account (Heglund and Cavagna, 1987; Lichtwark and Barclay, 2012; Lewis and Barclay, 2014). Note however, that in previous studies contraction conditions were chosen to maximize mechanical efficiency of concentric contractions. The contraction velocities imposed in the present study (0.4–0.8 resting muscle fiber lengths/s) are lower than those reported to elicit maximum mechanical efficiency in mouse soleus muscle (one fiber length/s, see Barclay et al., 2010; Table 1 therein). As such, it is conceivable that the maximum mechanical efficiency attainable in the present setup is higher than the currently reported value, and would be reached at higher contraction velocities than currently imposed.

The large decline in mechanical work per cycle over time within each trial observed here suggests that bouts of a few contractions are likely not representative of steady state behavior. We suggest that the initial decline in mechanical power output within a trial is the result of net phosphocreatine splitting within the fibers (Nagesser et al., 1992). The second more gradual decrease in mechanical power output might be related to fatigue of fast oxidative fibers, although we did not observe a relation between the within trial power decline and the percentage of type II fibers. Another possible explanation for the within-trial decline in mechanical power might be a reduced excitability of the muscle fibers. We did not check for the latter due to restrictions on the amplitude of the stimulus current in relation to electrolysis of the medium. However, the decline in mechanical power output was continuous rather than stepwise, the latter would be expected when fibers became unexcitable. Regardless of the source of the decline in mechanical power output, it highlights the need for prolonged measurement in order to capture all relevant aspects of repetitive contractions.

An important point to note is that in almost all previous work direct calorimetry was employed to quantify isolated muscle’s metabolic energy expenditure, whereas in the present study oxygen consumption was measured. The similarity in quantitative results observed between the present study and previous work thus lends mutual confidence to the validity of both methodologies, and to the verity of the results.

### Difference Between Human and Mouse Mechanical Efficiency

The concentric mechanical efficiency of mouse soleus muscle reported here and elsewhere in literature is lower than what is usually reported for submaximal exercise in intact humans (e.g., ∼0.22 during cycling Abbott et al., 1952, cf the discussion in Smith et al., 2005, pp. 29–30). There are two possible explanations for this discrepancy. Either the difference arises as an artifact of the experimental setup, or the mechanical efficiency of human muscle is truly higher than that of mouse muscle. Even though our results are in line with previous reports (as discussed above), experimental differences between the *in vivo* and *in vitro* situation may contribute to the difference in mechanical efficiency. However, in this study experimental conditions (stimulus frequency and movement pattern) were chosen to mimic mouse walking. James et al. (1995) reported that the soleus muscle in walking mice mostly acts as a stabilizing muscle, rather than generating substantial work. Consequently, both strain amplitude (∼5%) and contraction velocity (0.5–1.5 fiber optimum length/s, assuming a maximum contraction velocity of 4.8 optimum fiber length/s) are relatively small in walking mice (Table 2, in James et al., 1995). The present muscle strain (4–8% of muscle fiber resting length) and contraction velocity at peak force (0.4–0.8 resting fiber length/s) were close to these values reported for *in vivo* locomotion. Moreover, muscle temperature was kept close to the physiological temperature in this experiment. Given this similarity between the experimental conditions here and the *in vivo* case, a basic feasibility check for the measurement of mechanical efficiency at the muscle level is a comparison to whole body mechanical efficiency of ambulant mice – the former should be lower than the latter. However, for walking mice, it is difficult to obtain a baseline-subtracted efficiency, as resting metabolism in mice is relatively high (Schmidt-Nielsen, 1970), and the change in resting metabolism with exercise is unknown. Taylor et al. (1972) showed that the gross (i.e., not baseline subtracted) mechanical efficiency of mice walking on an inclined treadmill depends on vertical speed, with a maximum of 0.04 at a vertical speed of 0.14 m/s. Compared to this value, the value of ∼0.15 found here is certainly possible. In sum, we consider it unlikely that a difference between *in vivo* and *in vitro* mechanical efficiency arises as an artifact of the experimental conditions in this study.

Irrespective of the cause, it remains a challenge to reconcile the difference between human and mouse mechanical efficiency. In addition to the discussion put forth in Nelson et al. (2011), we note that three possibilities should be considered: (i) the efficiency of the contractile process is different; (ii) the metabolic energy required for activation is different (e.g., the efficiency of Calcium pumping), or (iii) the efficiency of ATP synthesis in the mitochondria is different. Given their smaller volume to surface area ratio, mice require – and indeed have – a higher resting metabolism per unit body mass (Schmidt-Nielsen, 1970). This higher resting metabolic rate may well be related to a lower mitochondrial efficiency. Option (iii) is thus a likely candidate hypothesis to explain the difference between mouse and human muscular mechanical efficiency.

## Conclusion

The amount of metabolic energy required for muscle activation was measured during physiologically relevant conditions in a mammalian preparation. The absolute amount of metabolic energy required for activation was found to be substantial and no clear relationship with contraction type was observed. The relative amount of metabolic energy required for activation was larger for eccentric than for concentric contraction. Net mechanical efficiency in contracting mouse soleus muscle during prolonged contractions ranged from 0.12 to 0.16, which is in line with previously reported values in which direct calorimetry was employed. We stress the importance of the periodic, prolonged nature of the contractions investigated here, as these may reveal important aspects of muscle operation *in vivo*, for example during locomotion. The results reported in this study will be used to further develop and validate models of muscle energetics in future work.

## Ethics Statement

This study was carried out in accordance with the recommendations of the VU University Animal Experimentation Ethical committee. The protocol was approved by the VU University Animal Experimentation Ethical committee (Protocol Number: DEC FBW1101).

## Author Contributions

KKL, RTJ, DAK, KvS, and WvdL conceived and designed the work. KKL, RTJ, DAK, KvS, and WvdL acquired, analyzed, and interpreted the data. KKL, RTJ, DAK, KvS, and WvdL drafted and revised the manuscript.

## Conflict of Interest Statement

The authors declare that the research was conducted in the absence of any commercial or financial relationships that could be construed as a potential conflict of interest.

## Figures and Tables

**APPENDIX A1:**
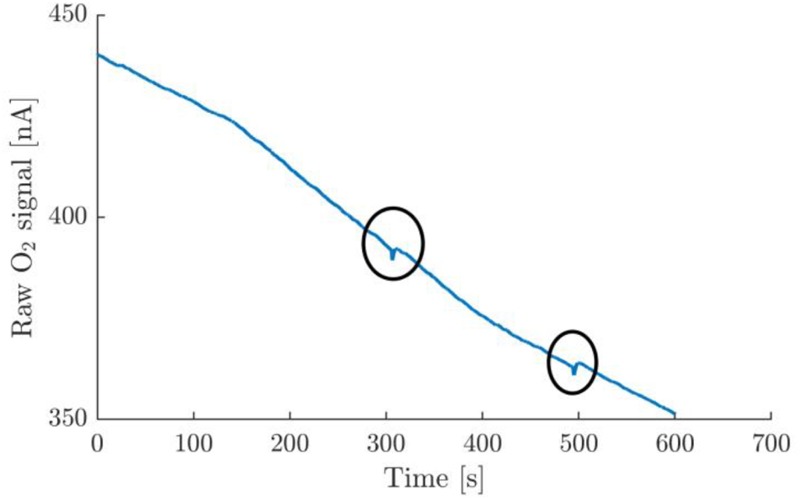
Example of a raw oxygen trace containing artifacts encircled. This type of disturbance in the signal was typical for trials which were deemed invalid and was readily observable in the signal. The most likely cause for this artifact is small gas bubbles which may have been trapped in the chamber during the assembly process, and which interact with the oxygen electrode, which protrudes the chamber at the very top.
